# Assessment of quality of life and cognitive dysfunction among patients with Major Depressive Disorder

**DOI:** 10.1192/j.eurpsy.2023.1769

**Published:** 2023-07-19

**Authors:** L. Abbasi, T. Mazzawi, T. Daradkeh

**Affiliations:** 1 Faculty of Medicine, Al-Balqa Applied University, Salt; 2 Jordan University of Science and Technology, Irbid, Jordan

## Abstract

**Introduction:**

Major Depressive Disorder (MDD) is associated with high mortality, disability and morbidity. Studies demonstrated mixed results on effects of depression treatments on quality of life (QOL).

**Objectives:**

To evaluate the severity of depression among Jordanian patients diagnosed with MDD before and after treatment and to find any relationship between QOL, depression severity and perceived cognitive dysfunction.

**Methods:**

Patients from both genders, 18-65 years old and diagnosed with MDD were included to attend two visits; at baseline and 6 weeks after treatment, in each they completed three questionnaires: Patient Health Questionnaire (PHQ-9) for depression severity, patient-rated Perceived Deficit Questionnaire (PDQ-5) for cognitive function, and World Health Organization Quality of Life Brief (WHOQOL-BREF) format.

**Results:**

A total of 92 patients completed the study. The scores of the different questionnaires and their correlations before and after treatment are presented in tables 1 and 2. Correlations between PHQ-9 and PDQ-5 before and after treatment are illustrated in figs 1 and 2, respectively.

**Table 1:** Total scores for WHOQOL-BREF format, PDQ-5 and PHQ-9, before and after treatment

**Questionnaire**	**Before**	**After**	***P*-value**
General quality of life	2.5±1	3.5±0.7	<0.0001
General health satisfaction	2.3±1	3.4±0.9	<0.0001
Physical health (Domain 1)	10±2.6	13.9±2.6	<0.0001
Psychological (Domain 2)	8±2.3	12.6±2.3	<0.0001
Social relationships (Domain 3)	9±3.2	12.8±2.8	<0.0001
Environment (Domain 4)	12.3±3	14.4±7.5	0.0003
Patient-rated Perceived Deficit 5 (PDQ-5)	13±4.4	7.2±4	<0.0001
Patient health questionnaire 9 (PHQ-9)	19±5.4	7.1±5.1	<0.0001

Values are presented as mean±SD. Analysis: Paired *t* test. P<0.05 is significant.

**Table 2:** Correlations between total scores for all of the domains for WHOQOL-BREF and PDQ-5, and between WHOQOL-BREF and PHQ-9, before and after treatment

**Domains/Facets**	**Patient-rated Perceived Deficit 5 (PDQ-5)**		**Patient health questionnaire 9 (PHQ-9)**	
	**Before (r, p)**	**After (r, p)**	**Before (r, p)**	**After (r, p)**
General quality of life	-0.38, 0.0001	-0.26, 0.01	-0.55, <0.0001	-0.49, <0.0001
General health satisfaction	-0.24, 0.02	-0.14, 0.2	-0.51, <0.0001	-0.48, <0.0001
Physical health (Domain 1)	-0.37, 0.0003	-0.50, <0.0001	-0.57, <0.0001	-0.76, <0.0001
Psychological (Domain 2)	-0.46, <0.0001	-0.43, <0.0001	-0.58, <0.0001	-0.68, <0.0001
Social relationships (Domain 3	-0.37, 0.0002	-0.37, 0.0002	-0.42, <0.0001	-0.40, <0.0001
Environment (Domain 4)	-0.52, <0.0001	-0.21, 0.047	-0.36, 0.0004	-0.12, 0.2

Spearman correlation (r); P<0.05 is significant. WHOQOL: World Health Organization Quality of Life.

**Image:**

**Figure:**
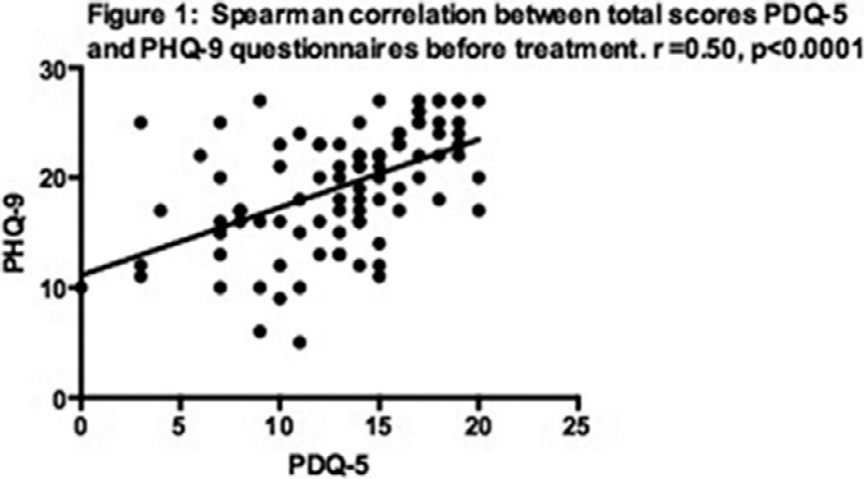

**Image 2:**

**Figure:**
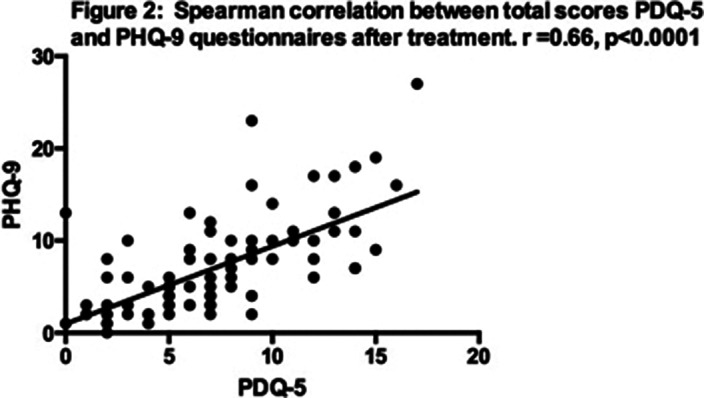

**Conclusions:**

Significant improvements were found in the symptoms of depression, cognition and QOL in patients with MDD after treatment. Depression severity significantly inversely correlated with QOL and cognition of MDD patients.

**Disclosure of Interest:**

None Declared

